# Hormesis: Calabrese Responds

**DOI:** 10.1289/ehp.0901681R

**Published:** 2010-04

**Authors:** Edward J. Calabrese

**Affiliations:** Environmental Health Sciences Division, School of Public Health and Health Sciences, University of Massachusetts, Amherst, Massachusetts, E-mail: edwardc@schoolph.umass.edu

In his letter, Mushak revisits his criticism (Mushak 2009) of previously reported hormesis frequency estimates (Calabrese and Baldwin 2001, 2003; Calabrese et al. 2006, 2008). In my commentary (Calabrese 2009), I addressed and/or rebutted in considerable detail his arguments (Mushak 2009), and no new data require me to revise that response. Here I address the key areas raised by Mushak’s letter, two of which relate to the frequency of hormesis, and the third considers the acceptance of hormesis by the scientific and regulatory communities.

First, a central point of Mushak’s commentary (Mushak 2009) and his letter is his assertion that the reported hormesis frequency of 37% (Calabrese and Baldwin 2001) is incorrect and should be 11%. Unfortunately, Mushak used the wrong denominator in his commentary, and he perpetuates this error in his letter. Briefly, we (Calabrese and Baldwin 2001) estimated the frequency of hormesis using *a priori* entry and evaluative criteria; some 668 dose responses satisfied the entry criteria. There were three independent evaluative criteria (i.e., hypothesis testing, nonoverlapping 95% confidence intervals, and alternative quantitative criteria). Of the 668 dose responses, 213 (31.8%) involved hypothesis testing. Of this total, 74 (74/213; 34.7%) satisfied the evaluative criteria for hormesis, a percentage similar to the other two evaluative approaches. When totaled, the three approaches yielded the 37% estimate. Mushak’s error is that he used the 74 dose responses that satisfied the evaluative criteria for hypothesis testing not only against the 213 dose responses that had hypothesis testing (which would have been a correct approach) but against all 668 dose responses, even though the remaining 455 dose responses that satisfied the entry criteria lacked hypothesis testing. None of these 455 dose responses could have been evaluated by the statistical criteria. Nonetheless, Mushak combined all the dose responses that satisfied the entry criteria and derived a hormesis frequency based on only dose responses with statistical significance. In so doing, he mistakingly reduced the 37% frequency to 11%. His method is the equivalent of using a raw score for the math component of the Graduate Record Examination (GRE) as the only source of correct answers, and then using all the questions on the math, verbal, and analytic components of the exam as the denominator, even though the student did not take these other components of the test. Such a calculation would give a useless GRE score. His method of hormesis calculation is clearly why he obtained the incorrect lower frequency.

Second, in his leter Mushak continues to cite a letter by Crump (2007) for which there is no support in the literature; also, Crump’s letter is based on an assumption about methods that was refuted by the National Cancer Institute investigators who actually did the original work (Calabrese et al. 2007). Mushak apparently does not grasp that Crump’s exercise inappropriately introduced 8-fold more variability into the data analysis. In his leter, Mushak incorrectly and inexplicably claimed that Crump’s analysis resulted in my conceding that the hormetic responses that we reported were not different from control responses.

Third, Mushak’s inflexibility concerning hormesis is reflected in his comments that minimize the impact of hormesis and its growing applications. Despite the significant biomedical impact of hormesis, Mushak fails to acknowledge the reality that hormetic effects are the basis for how most anxiolytic (Calabrese 2008a), antiseizure (Calabrese 2008b), memory (Calabrese 2008c; Zoladz and Diamond 2009), Alzeheimer disease (Calabrese 2008c; Congdon et al. 2009), and numerous other classes of drugs work (Kastin and Pan 2008; Mattson 2008; Sonneborn 2008; Thong and Maibach 2008), with all such drugs having to pass the regulatory oversight of the Food and Drug Administration for efficacy and safety. On the environmental side, Mushak—in both his letter and his commentary (Mushak 2009)—did not acknowledge that the largest-ever rodent cancer bioassay (24,000 mice) that was designed to determine the nature of the dose response in the low-dose zone for carcinogens revealed hormetic responses for acetyl aminofluorene- induced bladder cancer and that this was affirmed by the 14-member Society of Toxicology expert panel convened to assess these findings (Society of Toxicology ED_01_ Task Force 1981). In both his letter and his commentary (Mushak 2009), he also failed to acknowledge that hormesis has had a meteoric rise in recognition and journal citations within the scientific community, with 15 citations per year in the 1980s to > 2,400 in 2009 alone.

On these grounds and those presented in my commentary (Calabrese 2009), I conclude that Mushak’s arguments are without merit.
